# Polytherapy versus monotherapy in the treatment of tibial non-unions: a retrospective study

**DOI:** 10.1186/s10195-024-00763-5

**Published:** 2024-04-18

**Authors:** Fangzhou Lu, Rald V. M. Groven, Martijn van Griensven, Martijn Poeze, Jan A. P. Geurts, Shan Shan Qiu, Taco J. Blokhuis

**Affiliations:** 1 Division of Trauma Surgery, Department of Surgery, Maastricht University Medical Center, P. Debyelaan 25, 6229 HX Maastricht, the Netherlands; 2 Department of Cell Biology-Inspired Tissue Engineering, MERLN Institute for Technology-Inspired Regenerative Medicine, Maastricht University, Universiteitssingel 40, 6229 ER Maastricht, the Netherlands; 3 CAPHRI Care and Public Health Research Institute, Department of Orthopedic Surgery, Maastricht University Medical Centre, P. Debyelaan 25, 6229 HX Maastricht, Maastricht, the Netherlands; 4 Division of Plastic Surgery, Department of Surgery, Maastricht University Medical Center, P. Debyelaan 25, 6229 HX Maastricht, the Netherlands

**Keywords:** Tibial non-union, Polytherapy, Monotherapy, NUSS, RUST

## Abstract

**Background:**

Treating tibial non-unions efficiently presents a challenge for orthopaedic trauma surgeons. The established gold standard involves implanting autologous bone graft with adequate fixation, but the addition of biologicals according to the so-called diamond concept has become increasingly popular in the treatment of non-unions. Previous studies have indicated that polytherapy, which involves implanting mesenchymal stem cells, bioactive factors and osteoconductive scaffolds, can improve bone healing. This study aims to evaluate the efficacy of polytherapy compared with monotherapy in treating tibial non-unions of varying severity.

**Materials and methods:**

Data from consecutive tibial non-unions treated between November 2014 and July 2023 were retrospectively analysed. The Non Union Scoring System (NUSS) score before non-union surgery, and the Radiographic Union Score for Tibial fractures (RUST), scored at 1, 3, 6, 9, 12 and 18 months post-surgery, were recorded. Initially, a comparison was made between the polytherapy and monotherapy groups. Subsequently, patients receiving additional surgical non-union treatment were documented, and the frequency of these treatments was tallied for a subsequent per-treatment analysis.

**Results:**

A total of 34 patients were included and divided into a polytherapy group (*n* = 15) and a monotherapy group (*n* = 19). The polytherapy group demonstrated a higher NUSS score (44 (39, 52) versus 32 (29, 43), *P* = 0.019, *z* = −2.347) and a tendency towards a higher success rate (93% versus 68%, *P* = 0.104) compared with the monotherapy group. For the per-treatment analysis, 44 treatments were divided into the polytherapy per-treatment group (*n* = 20) and the monotherapy per-treatment group (*n* = 24). The polytherapy per-treatment group exhibited a higher NUSS score (48 (43, 60) versus 38 (30, 50), *P* = 0.030, *z* = −2.173) and a higher success rate (95% versus 58%, *P* = 0.006) than the monotherapy per-treatment group. Within the monotherapy per-treatment group, the NUSS score displayed excellent predictive performance (AUC = 0.9143). Setting the threshold value at 48, the sensitivity and specificity were 100.0% and 70.0%, respectively.

**Conclusions:**

Polytherapy is more effective than monotherapy for severe tibial non-unions, offering a higher success ratio. The NUSS score supports decision-making in treating tibial non-unions.

**Level of evidence:**

Level III.

**Supplementary Information:**

The online version contains supplementary material available at 10.1186/s10195-024-00763-5.

## Introduction

Bone non-union occurs in 5–10% of fracture patients, imposing physical, mental and financial burdens on individuals [1–5]. The tibia is one of the most frequently affected sites of bone non-union, with approximate incidence rates of 1.1% in non-operative treatment and 5% in surgically treated tibial fractures [6, 7]. Various risk factors that impede bone healing have been extensively documented, including delay in weight bearing, diabetes, osteoporosis, ageing, decreased oestrogen levels in postmenopausal females, smoking, alcohol consumption and use of non-steroidal anti-inflammatory drugs (NSAIDs), among others [8, 9]. Given the multifactorial aetiology, effectively treating tibial non-unions remains a significant challenge for orthopaedic trauma surgeons.

Currently, the established approach for addressing tibial non-unions is implanting autologous bone graft (ABG) in combination with adequate fixation [10, 11]. This surgical method is widely recognized as the gold standard for treating non-unions, including those at the tibial site [10, 11]. Nonetheless, the donor-site comorbidities associated with ABG implantation must still be taken into account, including the risk of infection, prolonged drainage, pain and potential sensory loss [11, 12]. Moreover, relying solely on ABG treatment for tibial non-union may not yield satisfactory results, even after multiple operative interventions [11, 13]. As a result, an increasing number of clinicians are looking for more effective approaches for treating tibial non-unions [11, 14, 15].

A decade ago, the ‘diamond concept’ was established to highlight crucial factors for successful bone healing, including osteoconductive scaffolds, osteogenic cells, mechanical stability, growth factors and vascularity [13, 16, 17]. Consequently, surgeons have begun to add mesenchymal stem cells (MSCs), bioactive factors and osteoconductive scaffolds to ABG implantation, or even apply them without ABG. The combination of these factors is referred to as polytherapy [17, 18], as opposed to monotherapy in which only ABG or one of these bioactive components is used.

Previous studies have indicated that polytherapy could be beneficial as compared with monotherapy in patients with more severe non-union, but these studies did not compare polytherapy and monotherapy in tibial non-unions [14, 15, 19–22]. Furthermore, implementation of a polytherapy approach potentially involves higher healthcare costs. Because of this, the role of polytherapy, especially in tibial non-unions, is still not clear. We conducted a retrospective analysis to investigate the effectiveness of polytherapy in comparison with monotherapy for the treatment of tibial non-unions with varying degrees of severity.

## Materials and methods

### Study design

The present retrospective clinical study was conducted at the Maastricht University Medical Center+ (the Netherlands), utilizing a patient database comprising consecutive individuals treated for tibial non-union from November 2014 to July 2023. The objective of this study was to evaluate the efficacy of the applied therapeutic strategy for addressing tibial non-unions. Specifically, we comprehensively compared the polytherapy approach with the monotherapy approach.

Recently, the Non Union Scoring System (NUSS) score was introduced to provide a comprehensive assessment of all potential risk factors contributing to the development of non-union [23, 24]. Accordingly, we utilized the NUSS score to evaluate the severity of non-unions, comparing two similar populations receiving either monotherapy or polytherapy. For assessing treatment outcomes, we employed the Radiographic Union Score for Tibial fractures (RUST) score [25].

### Patients and procedures

All procedures involving human participants were in accordance with the local Bioethics Committee and with the Helsinki Declaration (as revised in 2013). Inclusion criteria were as follows: (1) meeting the Food and Drug Administration (FDA) diagnostic criteria for non-union: a fracture that persists for a minimum of 9 months without signs of healing for 3 months [26], (2) age ≥ 18 years and (3) surgical treatment for a tibial non-union in our hospital. Exclusion criteria were as follows: (1) history of bone cancer, (2) pregnancy, (3) immunosuppressive drug therapy, (4) autoimmune disease and (5) neoplasia.

The data from a total of 34 patients met the inclusion criteria and were therefore analysed. Several data were retrieved from the electronic patient records. Demographic data, the type of non-union including the NUSS score, the index treatment and any subsequent treatments were recorded. Regarding the type of intervention, several biological materials are applied in our clinic, including ABG, reaming irrigation aspirator (RIA, Johnson and Johnson, USA), iFactor (Cerapedics, Westminster, CO, USA), Cerasorb (Curasan GmbH, Kleinostheim, Germany), bone marrow aspirate concentrate (BMAC, Arthrex, Naples, FL, USA), tricalcium phosphates (TCP), bone morphogenetic protein-2 (BMP-2, Medtronic, Minneapolis, MN, USA) and Polycaprolactone-tricalcium phosphate (PCL-TCP) three-dimensional (3D)-printed cages (Osteopore, Singapore). Each of these can be categorized into three distinct categories, namely MSCs, bioactive factors and osteoconductive scaffolds. Additionally, for several patients, BioActiveGlass (BAG S53P4, BonAlive, Turku, Finland) was utilized for its antibacterial properties rather than for osteoconduction. Consequently, bioglass was not classified under any specific category. In this study, monotherapy was defined as utilizing a maximum of one category out of the three types, while polytherapy involved a minimum of two out of three categories.

Firstly, we compared the outcome of the initial non-union surgical treatments between the monotherapy group (*n* = 19) and polytherapy group (*n* = 15) using the NUSS score and RUST score. Following the criteria of the RUST score, cases with a minimum score of 10 and no further non-union surgical treatment were scored as having a successful outcome. Cases with a maximum RUST score of 9 at the last follow-up visit, or cases that underwent additional surgical interventions, were considered to have a failure outcome.

Patients who received additional surgical treatment for their non-union were recorded as well. If the time between the first procedure and the additional treatment exceeded 6 months, the additional treatments were counted and a per-treatment analysis was performed accordingly. In this manner, one patient may contribute to multiple treatment categories, for example, if the first procedure consisted of a monotherapy treatment followed by polytherapy more than 6 months later. The same standardization of success and failure outcome was used to compare monotherapy per-treatment and polytherapy per-treatment. The RUST scores at 1, 3, 6, 9, 12 and 18 months post treatment were recorded, if available.

### Statistical analysis

Quantitative data are presented as either mean ± standard deviation (SD) or median (*P*_25_, *P*_75_), depending on whether the data followed a normal distribution as determined by the Shapiro–Wilk test. According to data characteristics, continuous quantitative data were compared between the two groups using Student’s *t*-test or the Mann–Whitney *U* test. Categorical variables were analysed using the chi-squared and Fisher’s exact tests. For evaluating potential imbalance of risk factors, multiple regression models were performed to adjust comparisons. Outcomes were recorded in binary format (‘success’ or ‘failure’). In the comparison between the polytherapy per-treatment group and the monotherapy per-treatment group, the two groups under examination are no longer entirely independent, as they partially share several patients. Consequently, a mixed linear model was used to analyse this non-independence to compare the treatment outcome between each group. RUST scores were examined both as continuous variables and in binary form (‘RUST score ≥ 10’ or ‘RUST score < 10’) at 1, 3, 6, 9, 12 and 18 months post treatment. We evaluated the association between treatment types and overall treatment outcome in a Cox proportional hazards regression model. We conducted binary logistic regression analysis to investigate the influence of NUSS score on the outcomes of monotherapy patients. The predictive capability of NUSS was assessed using the area under the ROC curve (AUC) with a criterion of maximizing the sum of sensitivity and specificity as the diagnostic threshold. A two-sided test was employed, and statistically significant difference was defined as *P* value < 0.05. Data analysis was conducted using SPSS 27 (SPSS Statistics for Windows, version 27.0.0; IBM, Armonk, NY, USA), while survival analysis was performed using GraphPad Prism 10 (Prism for Windows, version 10.0.2; GraphPad Boston, MA, USA).

## Results

### Comparison of the polytherapy group and monotherapy group

**Fig. 1:**
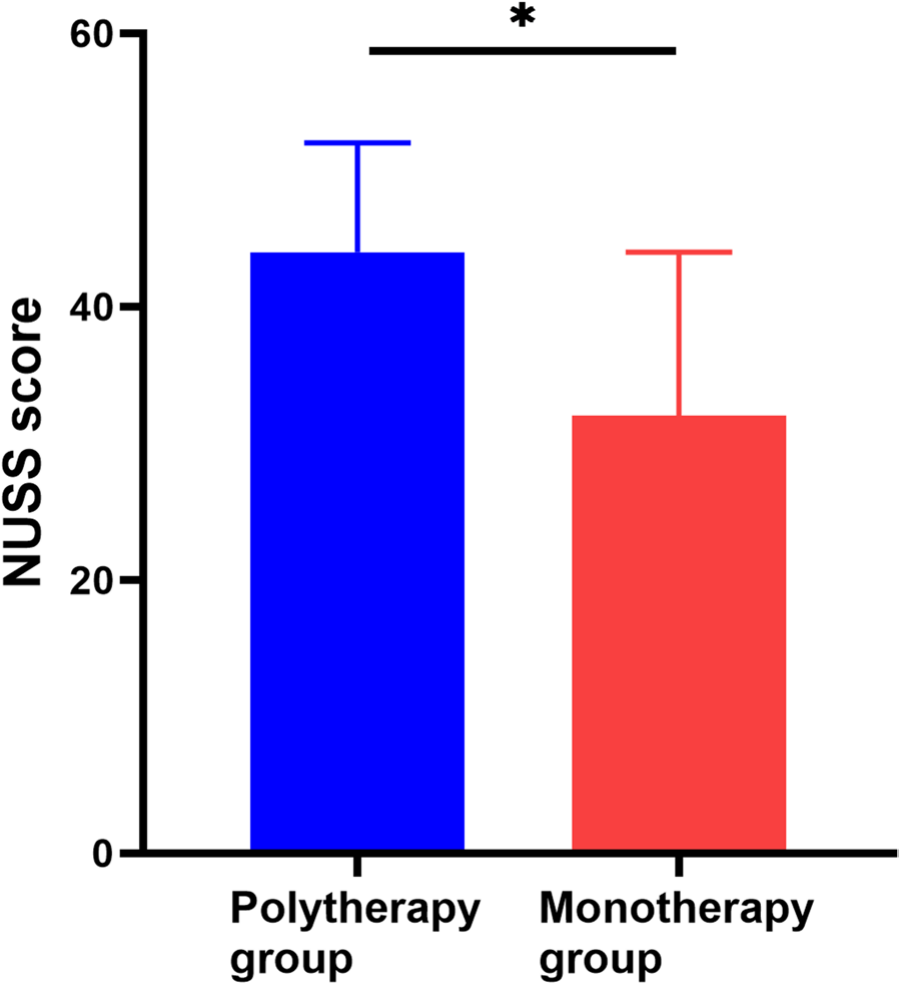
Comparison of NUSS scores between the polytherapy group and the monotherapy group. * *P* < 0.05

The demographic characteristics of the polytherapy group and monotherapy group are summarized in Table 1. The baseline demographic parameters are similar for age, sex distribution, body mass index (BMI), fixation strategy, incidence of isolated fractures, smoking status, presence of hypertension, diabetes mellitus, hypercholesterolaemia, respiratory disease, congestive heart failure, coronary artery disease, prior cerebrovascular event, liver disease, cancer history in other systems and peripheral vascular disease. The polytherapy group demonstrated a statistically higher NUSS score (44 (39, 52) versus 32 (29, 43), *P* = 0.019, *z* = −2.347) compared with the monotherapy group (Fig. 1). The multiple regression analysis described that age, NUSS score, high-energy trauma and fracture type do not significantly interfere with the relationship between the independent variable and the dependent variable (Additional file 1: Table S1). Furthermore, the polytherapy group demonstrated a significantly higher percentage of high-energy trauma cases (67% versus 26%, *P* = 0.036) and a trend towards a higher level of Gustilo classification compared with the monotherapy group (*P* = 0.066, *z* = −2.001).

**Table 1:** Demographic characteristics of the polytherapy group and monotherapy group

	Polytherapy (*n* = 15)	Monotherapy (*n* = 19)	*P* value
Age (*M*(*P*_25_, *P*_75_)) (years)	47 (36, 57)	60 (47.5, 62)	0.06 (*z* = −1.875)
Female (%)	4 (27%)	7 (37%)	0.715
BMI (Mean ± SD) (weight (kg)/height (m)^2^)	24.6 ± 0.8	27.0 ± 1.2	0.139
NUSS score (*M* (*P*_*25*_, *P*_*75*_))	44 (39, 52)	32 (29, 43)	0.019 (*z* = −2.347)^*^
High-energy trauma cases (%)	10 (67%)	5 (26%)	0.036^*^
Fracture type (0: I: II: IIIA: IIIB: IIIC)^a^	5:2:3:3:2:0	13:1:3:1:1:0	0.066 (*z* = −2.001)
Fixation (Intramedullary nail: plate: conservative)	7:5:3	7:12:0	0.891 (*z* = −0.155)
Isolated fracture (%)	11 (73%)	15 (79%)	1.000
Smoking status (%)	5 (33%)	3 (16%)	0.417
Hypertension (%)	0 (0%)	1 (5%)	1.000
Diabetes mellitus (%)	2 (13%)	1 (5%)	0.571
Hypercholesterolaemia (%)	0 (0%)	2 (11%)	0.492
Respiratory disease (%)	2 (13%)	4 (21%)	0.672
Congestive heart failure (%)	2 (13%)	3 (16%)	1.000
Coronary artery disease (%)	2 (13%)	2 (11%)	1.000
Prior cerebrovascular event (%)	0 (0%)	1 (5%)	1.000
Liver disease (%)	0 (0%)	0 (0%)	NA^b^
Cancer history in other systems (%)	2 (13%)	0 (0%)	0.187
Peripheral vascular disease (%)	0 (0%)	1 (5%)	1.000

^a^ Close fracture was recorded as ‘0’ in the present study. Gustilo open fracture classification: Type I: open fracture, clean wound, wound < 1 cm in length; Type II: open fracture, wound > 1 cm but < 10 cm in length without extensive soft-tissue damage, flaps, avulsions; type IIIA: open fracture with adequate soft-tissue coverage of a fractured bone despite extensive soft-tissue laceration or flaps, or high-energy trauma (gunshot and farm injuries) regardless of the size of the wound; Type IIIB: open fracture with extensive soft-tissue loss and periosteal stripping and bone damage, usually associated with massive contamination, will often need further soft-tissue coverage procedure (i.e. free or rotational flap); Type IIIC: open fracture associated with an arterial injury requiring repair, irrespective of degree of soft-tissue injury. ^b^ In both the monotherapy patients group and the polytherapy patients group, there are no cases with liver disease. Therefore, the *P* value is not applicable (NA). * *P* < 0.05

**Fig. 2:**
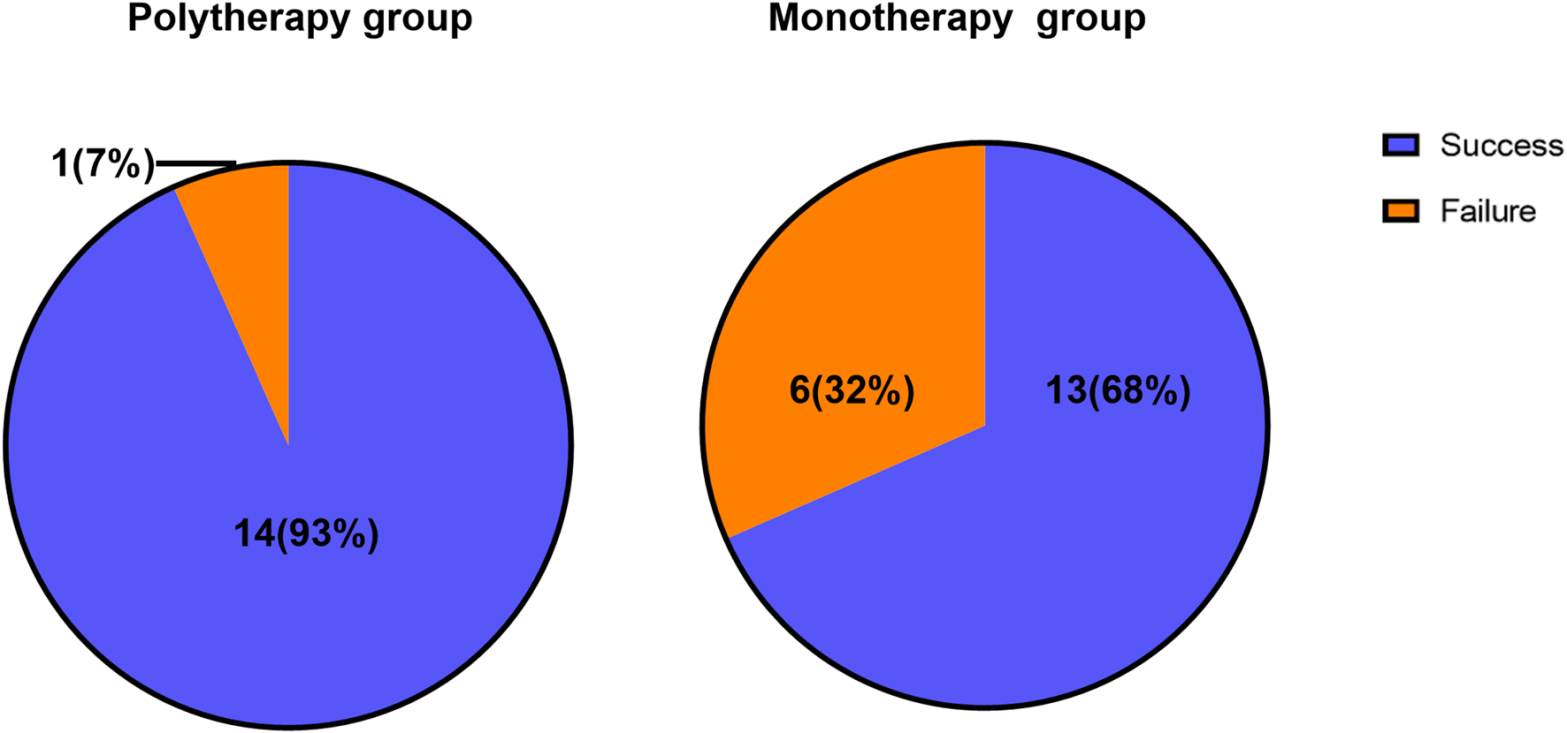
Success/failure outcomes of the polytherapy group and monotherapy group

Although the success rate in the polytherapy group was higher than in the monotherapy group, this difference was not statistically significant (93% versus 68%, *P* = 0.104) (Fig. 2). Of the 19 patients who received monotherapy in the index surgical procedure, six developed a failure and subsequently underwent additional surgical interventions. One of these patients developed an infection, for which revision at 3 months was necessary. Therefore, this patient was recorded as a failure with a follow-up of 3 months. All the other revisions were polytherapy treatments performed at 3 months (*n* = 1), 3 months (*n* = 1) and more than 1 year (*n* = 4) after the first procedure, and were subsequently included in the per-treatment analysis.

### Comparison of the polytherapy per-treatment group and monotherapy per-treatment group

According to the aforementioned description, we analysed data for each treatment from a cohort of 34 recruited patients. The demographic characteristics of both the polytherapy per-treatment group and the monotherapy per-treatment group are summarized in Table 2. Notably, these two groups exhibited several similarities in terms of age, sex distribution, BMI, fixation strategy, incidence of isolated fractures, smoking status, presence of hypertension, diabetes mellitus, hypercholesterolaemia, respiratory disease, congestive heart failure, coronary artery disease, prior cerebrovascular events, liver disease, cancer history in other systems and peripheral vascular disease.

**Table 2:** Demographics characteristics of the polytherapy per-treatment group and monotherapy per-treatment group

	Polytherapy per-treatment (*n* = 20)	Monotherapy per-treatment (*n* = 24)	*P* value
Age (Mean ± SD) (years)	47.4 ± 14.0	52.5 ± 13.3	0.223
Female (%)	5 (25%)	7 (29%)	0.514
BMI (Mean ± SD) (weight (kg)/height (m)^2^)	25.0 ± 4.0	27.3 ± 5.7	0.135
NUSS (*M* (*P*_*25*_, *P*_*75*_))^b^	48 (43, 60)	38 (30, 50)	0.030 (*z* = −2.173)^*^
High-energy trauma cases (%)	11 (55%)	6 (25%)	0.063
Fracture type (0: I: II: IIIA: IIIB: IIIC)^a^	7:2:5:3:3:0	14:1:5:1:3:0	0.170 (*z* = −1.372)
Fixation (Intramedullary nail: Plate: conservative)^c^	8:7:5	8:13:3	0.878 (*z* = −0.153)
Isolated fracture (%)	16 (80%)	20 (83%)	1.000
Smoking status (%)	7 (35%)	4 (17%)	0.185
Hypertension (%)	0 (0%)	1 (4%)	1.000
Diabetes mellitus (%)	2 (10%)	1 (4%)	0.538
Hypercholesterolaemia (%)	1 (5%)	4 (17%)	0.356
Respiratory disease (%)	3 (15%)	6 (25%)	0.477
Congestive heart failure (%)	2 (10%)	3 (13%)	1.000
Coronary artery disease (%)	2 (10%)	2 (8%)	1.000
Prior cerebrovascular event (%)	0 (0%)	1 (4%)	1.000
Liver disease (%)	0 (0%)	0 (0%)	NA^b^
Cancer history in other systems (%)	2 (10%)	0 (0%)	0.201
Peripheral vascular disease (%)	1 (5%)	3 (13%)	0.614

^a^ Close fracture was recorded as ‘0’ in the present study. Gustilo open fracture classification: Type I: open fracture, clean wound, wound < 1 cm in length; Type II: open fracture, wound > 1 cm but < 10 cm in length without extensive soft-tissue damage, flaps, avulsions; Type IIIA: open fracture with adequate soft-tissue coverage of a fractured bone despite extensive soft-tissue laceration or flaps, or high-energy trauma (gunshot and farm injuries) regardless of the size of the wound; Type IIIB: open fracture with extensive soft-tissue loss and periosteal stripping and bone damage, usually associated with massive contamination, will often need further soft-tissue coverage procedure (i.e. free or rotational flap); Type IIIC: open fracture associated with an arterial injury requiring repair, irrespective of degree of soft-tissue injury. ^b^ In both the monotherapy patients group and the polytherapy patients group, there are no cases with liver disease. Therefore, the *P* value is not applicable (NA). * *P* < 0.05

**Fig. 3:**
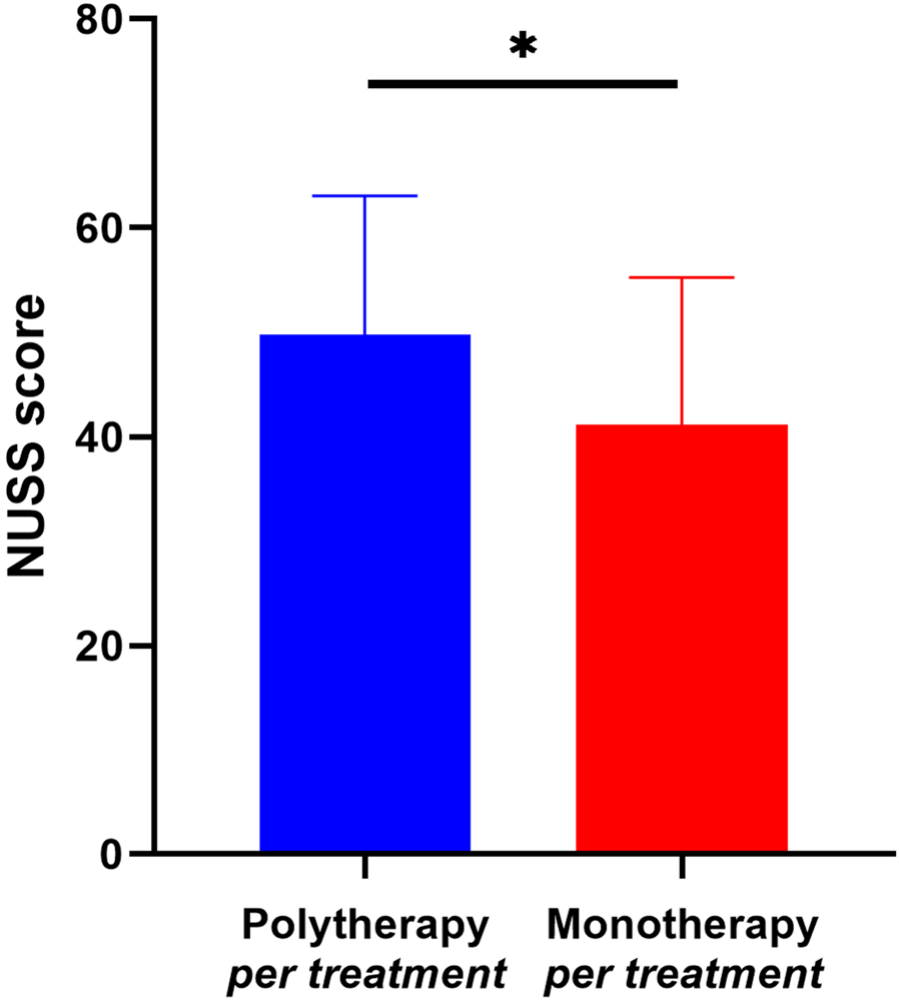
Comparison of NUSS scores between the polytherapy per-treatment group and monotherapy per-treatment group. * *P* < 0.05

In contrast to the previous demographic analysis, there was no statistically significant difference in the percentage of high-energy trauma cases between the polytherapy per-treatment group and the monotherapy per-treatment group (55% versus 25%, *P* = 0.063). The NUSS score was statistically higher in the polytherapy per-treatment group compared with the monotherapy per-treatment group (48 (43, 60) versus 38 (30, 50), *P* = 0.030, *z* = −2.173) (Fig. 3).

**Fig. 4:**
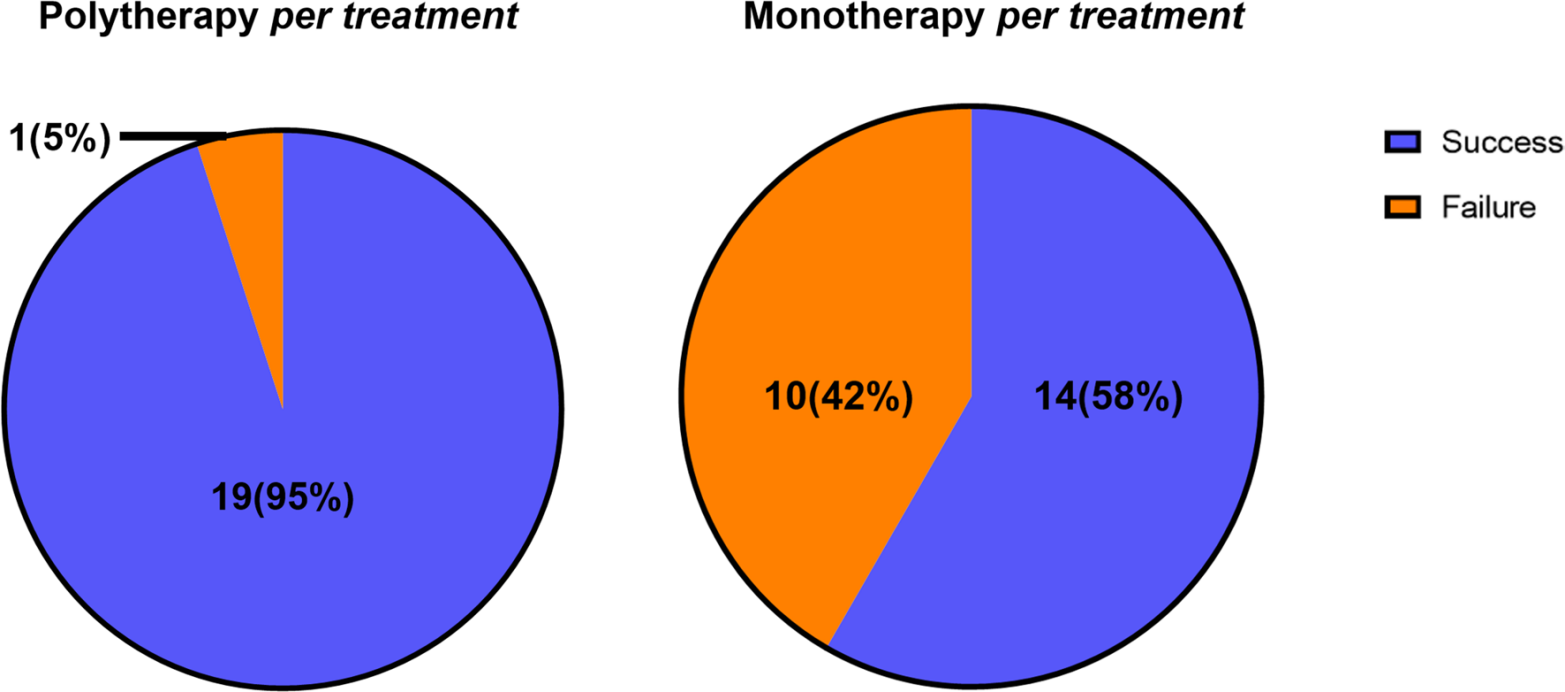
Success/failure outcomes of the polytherapy per-treatment group and the monotherapy per-treatment group

The success rate in the polytherapy per-treatment group was statistically higher than in the monotherapy per-treatment group (95% versus 58%, *P* = 0.006) (Fig. 4). Additionally, the mixed linear regression model analysis described that, when the NUSS score was fixed, the polytherapy per-treatment group statistically has a better treatment outcome than the monotherapy per-treatment group (Additional file 2: Table S2). When considered in conjunction with the NUSS score results, this suggests that, despite the greater severity of non-union in the polytherapy per-treatment group, polytherapy proves to be an effective approach in ultimately achieving a higher success rate.

**Fig. 5:**
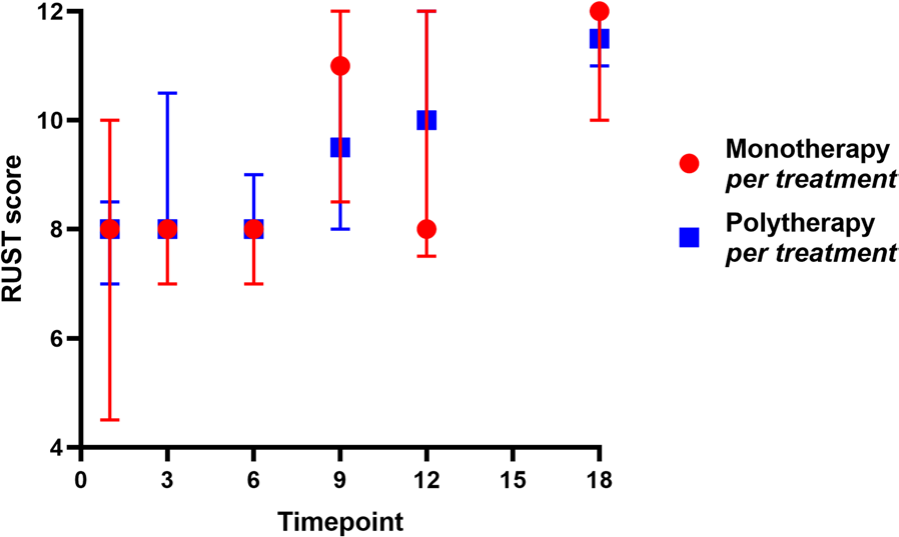
Cox proportional hazards regression model of RUST scores at each time point (1, 3, 6, 9, 12 and 18 months post surgery) for the polytherapy per-treatment group and monotherapy per-treatment group

**Fig. 6:**
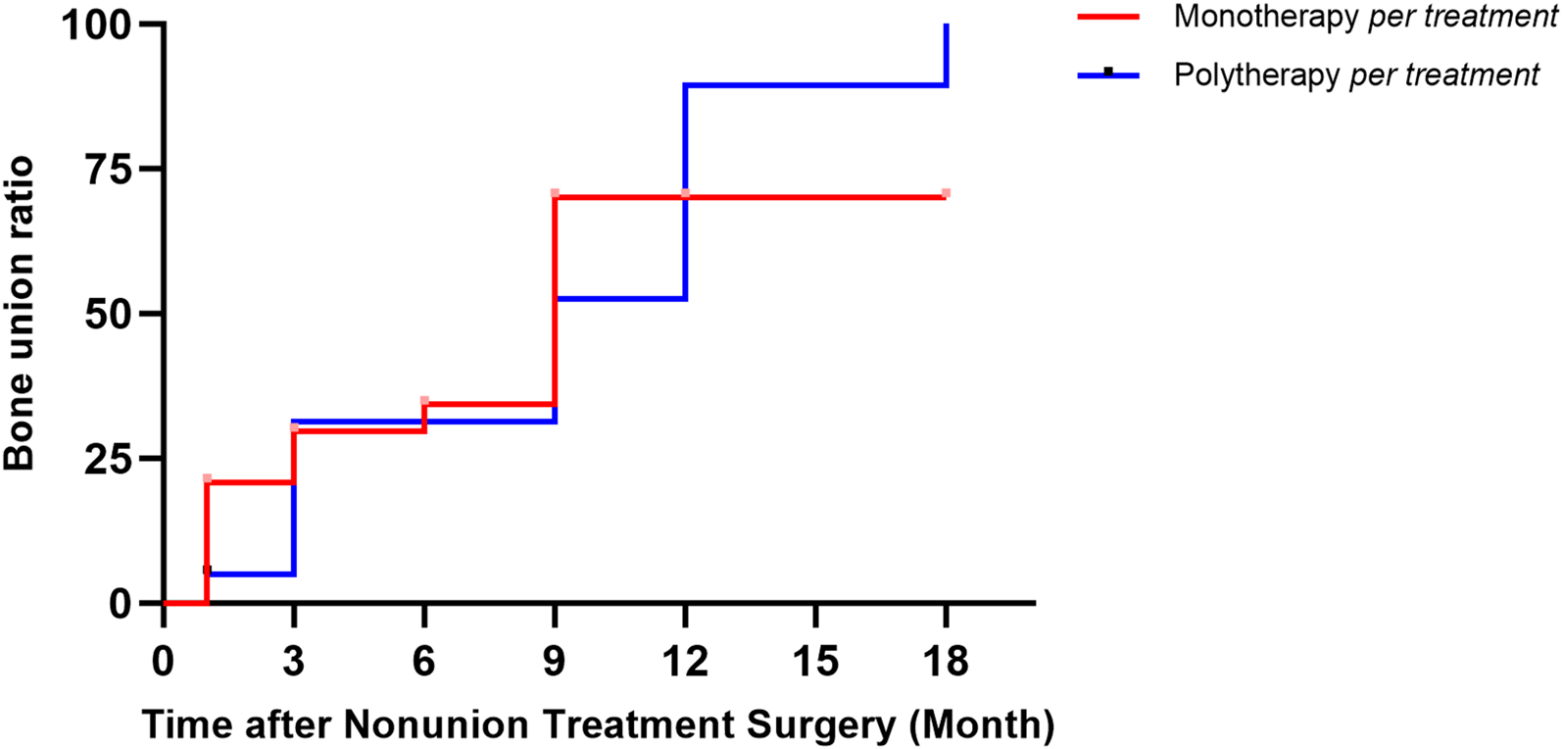
Survival analysis of the polytherapy per-treatment group and monotherapy per-treatment group

The RUST scores for each group at each time point (1, 3, 6, 9, 12 and 18 months post surgery) showed no statistically significant differences (Fig. 5). Similarly, the results of the survival analysis indicated no significant difference in bone healing ratios between the two groups (Fig. 6). Collectively, these findings support the idea that polytherapy is a reliable approach for addressing more severe cases of tibial non-union, yielding satisfactory outcomes.

**Fig. 7:**
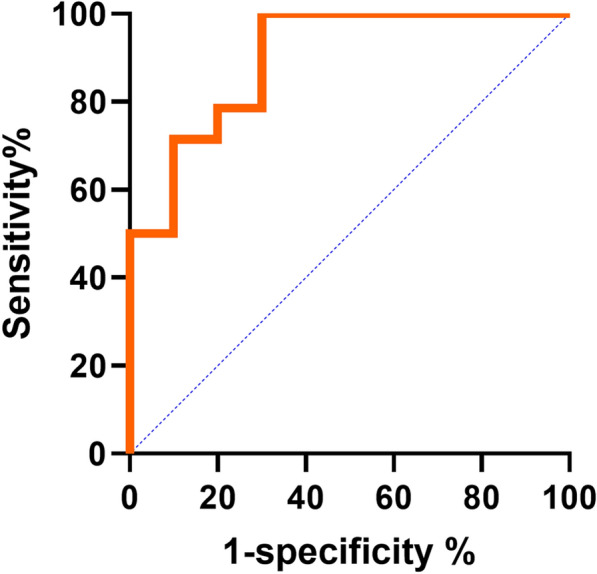
ROC curve analysis of NUSS score for predicting prognosis in monotherapy

The binary logistic regression analysis showed that the NUSS score serves as a predictor of prognosis in monotherapy (*P* < 0.001). The predictive performance of the NUSS score was evaluated through ROC analysis, yielding an AUC of 0.9143 (95% CI 0.8015–1.000) (*P* = 0.0007). With a threshold value set at 48, the sensitivity and specificity were 100.0% and 70.0%, respectively (Fig. 7). The analysis was not performed in the polytherapy per-treatment group owing to there being just one case with a failure outcome.

## Discussion

This retrospective analysis compared monotherapy with polytherapy in tibial non-unions and showed a higher success rate of 95% in patients treated with polytherapy despite a higher NUSS score in the polytherapy group, which indicates more severe non-unions. Moreover, failures in the monotherapy group were successfully treated using a polytherapy approach. This indicates that more severe non-unions benefit from a polytherapy treatment in which cells, scaffolds and growth factors are combined. Although this treatment strategy may appear complicated, the benefit for patients and the healthcare system is evident by avoiding secondary treatments.

Several publications have described the effectiveness of polytherapy treatment for non-unions [3, 14, 15, 27]; For example, a retrospective clinical study comparing monotherapy with polytherapy for forearm non-unions showed increased healing in the polytherapy group, despite having more severe non-unions, as reflected by a higher NUSS score. However, it remains ambiguous which non-union severity and anatomical location should dictate the choice for polytherapy, as e.g. forearm non-unions differ from tibial non-unions in terms of infection rate and soft-tissue coverage [28, 29].

We have presented several indicators supporting the effectiveness of polytherapy as a treatment, particularly for severe cases of tibial non-union, including a higher NUSS score. These findings align with the current consensus in other studies which support the effectiveness of polytherapy [3, 14, 15, 27]. It is worth noting that, in contrast to several other studies [14, 30], our polytherapy per-treatment group had no case involving autologous iliac crest grafts. Despite this, our polytherapy treatment still yielded a 95% success rate, surpassing the 90% success rate achieved with ABG treatment for non-union in a separate study [30].

To provide further clarity regarding the threshold for opting for polytherapy over monotherapy, we conducted a detailed analysis of the correlation between NUSS scores and outcomes within the monotherapy per-treatment group. In 2008, Calori et al. introduced a comprehensive evaluation system for bone non-unions, known as NUSS, which encompasses all risk factors influencing non-union [23]. NUSS indicates that patients with higher scores require more specialized care and treatment for non-union. Our findings, wherein the polytherapy per-treatment group exhibits both a higher NUSS score and a greater success ratio, align with this principle. To our knowledge, this is the first study to establish a direct connection between the severity of non-union, NUSS score values and treatment outcomes in tibial cases. It becomes apparent that monotherapy is suitable for patients with low NUSS scores, while polytherapy is beneficial for those with high NUSS scores.

Nevertheless, there remains ambiguity among orthopaedic surgeons in defining low and high values of NUSS scores for choosing polytherapy or monotherapy. To establish a quantifiable threshold, we focused on analysing the monotherapy per-treatment group. Our data revealed that a NUSS score of 48 can function as a prognostic indicator in monotherapy. This suggests that, when the NUSS score reaches 48, monotherapy may not be the optimal choice for treating tibial non-unions. It remains to be seen, however, whether patients with NUSS score below 48 benefit from polytherapy instead of monotherapy. In our cohort, six patients with failed monotherapy healed after polytherapy. Theoretically, the effect of the first surgical procedure (monotherapy) could have contributed to their healing, but considering the fact that the revision took place more than 1 year after the first treatment in four of the patients, this effect is very unlikely. Future studies should aim to provide further confirmation by expanding the patient cohort.

To evaluate the postoperative recovery period, we examined the healing ratio and RUST score values at various time points following surgery. Notably, an observable trend emerged: after 12 months post surgery, polytherapy demonstrated a higher percentage of success outcomes and elevated RUST scores. This indicates that polytherapy may potentially expedite recovery for patients, a finding that warrants further clarification in future, larger cohort studies.

Our study has several limitations. First, the retrospective nature of the study is inherent, limiting the availability of some data, such as the RUST score for each time point. Still, we included consecutive non-unions to avoid inclusion bias. Second, we did not examine the impact of infection on polytherapy or monotherapy outcome. The presence of infection is, however, embedded in the NUSS score to evaluate the severity of tibial non-union and is thus part of the analysis. Third, the sample size was constrained, although the observed difference was significant in the per-treatment analysis. However, our study benefits from meticulous statistical analysis and an extensive follow-up period, thereby increasing the validity of our data. It illustrates the effectiveness of polytherapy for tibial non-unions and indicates a threshold for choosing between polytherapy and monotherapy. These insights may guide surgeons in determining the most suitable treatment strategy for tibial non-union.

### Supplementary Information

**Additional file 1: Table S1. **Multiple regression models to adjust comparisons between polytherapy group and monotherapy group.

**Additional file 2: Table S2. **Mixed linear model for comparing the treatment outcome between polytherapy *per treatment* group and monotherapy *per treatment* group.

## Data Availability

The datasets used and/or analysed during the current study are available from the corresponding author on reasonable request.

## Abbreviations

NUSS: Non Union Scoring System
RUST: Radiographic Union Score for Tibial fractures
NSAIDs: Non-steroidal anti-inflammatory drugs
ABG: Autologous bone graft
MSCs: Mesenchymal stem cells
FDA: Food and Drug Administration
RIA: Reaming irrigation aspirator
BMAC: Bone marrow aspirate concentrate
TCP: Tricalcium phosphate
BMP-2: Bone morphogenetic protein-2
BAG: BioActiveGlass
SD: Standard deviation
BMI: Body mass index
