# Supplementary material for: Polytherapy versus monotherapy in the treatment of tibial non-unions: a retrospective study
Source: J Orthop Traumatol. 2024 Apr 18;25:21. doi: 10.1186/s10195-024-00763-5 (PMC11026327; doi:10.1186/s10195-024-00763-5)
Supplement: Supplementary file 1 — Additional file 1: Table S1. Multiple regression models to adjust comparisons between polytherapy group and monotherapy group. [file 10195_2024_763_MOESM1_ESM.docx]

**Additional file 1: TableS1:** Multiple regression models to adjust comparisons between polytherapy group and monotherapy group

|  | **B** | **S.E.** | **Wald** | **P** | **Exp(B)** | **95% C.I. for EXP(B)** | |
| --- | --- | --- | --- | --- | --- | --- | --- |
|  |  |  |  |  |  | **Lower** | **Upper** |
| Age | -0.061 | 0.033 | 3.366 | 0.067 | 0.941 | 0.882 | 1.004 |
| NUSS score | 0.045 | 0.043 | 1.092 | 0.296 | 1.046 | 0.962 | 1.137 |
| High-energy trauma | 1.101 | 0.924 | 1.418 | 0.234 | 3.006 | 0.491 | 18.394 |
| Fracture type (Gustilo) | 0.363 | 0.420 | 0.748 | 0.387 | 1.438 | 0.632 | 3.274 |

Note: The confounding factors age, NUSS score, High-energy trauma, and fracture type will not significantly interfere with the relationship between the independent variable and the dependent variable.
