# Supplementary material for: Polytherapy versus monotherapy in the treatment of tibial non-unions: a retrospective study
Source: J Orthop Traumatol. 2024 Apr 18;25:21. doi: 10.1186/s10195-024-00763-5 (PMC11026327; doi:10.1186/s10195-024-00763-5)
Supplement: Supplementary file 2 — Additional file 2: Table S2. Mixed linear model for comparing the treatment outcome between polytherapy per treatment group and monotherapy per treatment group. [file 10195_2024_763_MOESM2_ESM.docx]

**Additional file 2: Table S2:** Mixed linear model for comparing the treatment outcome between polytherapy *per treatment* group and monotherapy *per treatment* group

|  | **F** | **Sig.** |
| --- | --- | --- |
|  |  |  |
| Intercept | 34.055 | 0.000 |
| NUSS score | 1.486 | 0.180 |
| Group | 10.286 | 0.004 |

Note: Dependent Variable: Outcome. Fixing NUSS score in analysis indicates that polytherapy *per treatment* group statistically has a better treatment outcome than monotherapy *per treatment* group.
